# Reduced Proteasome Activity and Enhanced Autophagy in Blood Cells of Psoriatic Patients

**DOI:** 10.3390/ijms21207608

**Published:** 2020-10-14

**Authors:** Piotr Karabowicz, Adam Wroński, Halina Ostrowska, Georg Waeg, Neven Zarkovic, Elżbieta Skrzydlewska

**Affiliations:** 1 Department of Analytical Chemistry, Medical University of Bialystok, Mickiewicza 2D, 15-222 Bialystok, Poland; piotr.karabowicz@umb.edu.pl; 2 Dermatological Specialized Center “DERMAL” NZOZ in Bialystok, 15-453 Bialystok, Poland; adam.wronski@dermal.pl; 3 Department of Biology, Medical University of Bialystok, Mickiewicza 2D, 15-222 Bialystok, Poland; halina.ostrowska@umb.edu.pl; 4 Institute of Molecular Biosciences, University of Graz, 8010 Graz, Austria; georg.waeg@uni-graz.at; 5 LabOS, Laboratory for Oxidative Stress, Rudjer Boskovic Institute, Bijenicka 54, HR-1000 Zagreb, Croatia; zarkovic@irb.hr

**Keywords:** psoriasis, oxidative stress, proteasomal activity, immunoproteasomal subunits, autophagy

## Abstract

Psoriasis is a skin disease that is accompanied by oxidative stress resulting in modification of cell components, including proteins. Therefore, we investigated the relationship between the intensity of oxidative stress and the expression and activity of the proteasomal system as well as autophagy, responsible for the degradation of oxidatively modified proteins in the blood cells of patients with psoriasis. Our results showed that the caspase-like, trypsin-like, and chymotrypsin-like activity of the 20S proteasome in lymphocytes, erythrocytes, and granulocytes was lower, while the expression of constitutive proteasome and immunoproteasome subunits in lymphocytes was increased cells of psoriatic patients compared to healthy subjects. Conversely, the expression of constitutive subunits in erythrocytes, and both constitutive and immunoproteasomal subunits in granulocytes were reduced. However, a significant increase in the autophagy flux (assessed using LC3BII/LC3BI ratio) independent of the AKT pathway was observed. The levels of 4-HNE, 4-HNE-protein adducts, and proteins carbonyl groups were significantly higher in the blood cells of psoriatic patients. The decreased activity of the 20S proteasome together with the increased autophagy and the significantly increased level of proteins carbonyl groups and 4-HNE-protein adducts indicate a proteostatic imbalance in the blood cells of patients with psoriasis.

## 1. Introduction

Psoriasis is a chronic inflammatory autoimmune disease of the skin and joints. Its pathogenesis is thought to include interactions between genetic factors (for example, the *HLACw6* and *HLACw7* tissue compatibility complex alleles) and environmental factors such as bacterial or viral infections, injuries, and stress [1]. Psoriasis is mediated by T cells, dendritic cells, neutrophils, and other leukocytes. In psoriasis, inflammatory cytokines, such as interleukins IL-23 and IL-6, are produced by dendritic cells and macrophages. These cytokines facilitate the differentiation of Th17 cells, which secrete IL-17 and other mediators that stimulate the hyperproliferation of epidermal cells and contribute to abnormal keratinocyte differentiation [2]. In addition, proinflammatory signaling pathways such as MAPK/AP-1, NF-κB, and JAK-STAT are activated in cells involved in the development of psoriatic lesions [3,4,5].

One group of factors that modulate cell signaling pathways are reactive oxygen species (ROS), which are constantly generated as a result of normal cellular metabolism, but are produced at increased quantities as a result of exogenous factors and metabolic disorders [6]. Under physiological conditions, the effect of ROS is balanced by the activity of endogenous antioxidants. However, overproduction or prolonged action of ROS leads to oxidative stress and, as a result, oxidative modifications of biologically active cell components, including DNA, lipids, and proteins [7]. Many studies have revealed elevated levels of markers of oxidative damage in the serum/plasma and blood cells of patients with psoriasis [8,9,10,11,12,13,14]. Additionally, there is a positive correlation between oxidative stress markers and psoriasis area and disease severity index [15]. Recent reports have confirmed that psoriasis is accompanied by enhanced lipid peroxidation, demonstrated by the increased generation of α,β unsaturated reactive aldehydes (e.g., 4-HNE) that form adducts with proteins. These products may play an important role in the pathophysiology of psoriasis [11,12,16].

Under oxidative conditions, when the amount of oxidatively modified proteins increases, the activity of proteolytic systems (especially the 20S and 26S proteasome system and autophagy) is essential to ensure proteostasis. When the performance of these systems is disturbed, misfolded and aggregated proteins can accumulate to toxic levels and cause cell dysfunction, potentially leading to the development of many diseases [17]. However, as well as increasing the level of modified proteins, oxidative stress may also promote the increased activity of proteasomes. As evidence of this, it has been demonstrated under conditions of permanent oxidative stress, the 26S proteasome and the ubiquitinating system are deactivated [7]. The 20S proteasome degrades oxidatively modified proteins without ubiquitination [18]. Multiple studies from a number of laboratories have independently confirmed that the main pathway for the degradation of oxidatively damaged proteins in cells is via the 20S proteasome [19,20,21].

The 20S proteasome consists of α and β subunits. Three of the β subunits (β1, β2, β5) contain catalytic sites that perform various proteolytic activities [18]. These are classified as caspase-like (β1), trypsin-like (β2), and chymotrypsin-like (β5) as defined by the presence of their cleavage sites after acidic, basic, or hydrophobic amino acids, respectively [22]. However, after exposure of cells to proinflammatory factors (interferon-γ (IFN-γ) or tumor necrosis factor (TNFα)), three catalytic 20S subunits are replaced with homologous subunits, β1i (LMP2), β2i (MECL1), and β5i (LMP7) to form an immunoproteasome with enhanced chymotrypsin-like activity (β1i and β5i) and trypsin-like activity (β2i) [23]. The immunoproteasome is involved in the pathogenesis of inflammatory diseases [24] and is also responsible for degrading oxidatively damaged proteins. The biogenesis of the immunoproteasome is strongly induced during adaptation to oxidative stress [25], and it is constitutively expressed in cells of lymphoid origin [26].

In addition to the degradation of oxidized proteins, the proteasome is involved in the regulation of many other signaling pathways, including cell differentiation, proliferation, and apoptosis, as well as transcription activation and angiogenesis [27]. One of the best-studied roles of an immunoproteasome is in antigen presentation, and immunoproteasome dysfunction can lead to numerous immune and inflammatory diseases [28]. The development of psoriasis has been linked with allelic variation in β1i, and β5i 20S proteasome subunits [29]. The expression and activity of the 20S proteasome are also increased in psoriatic skin cells [30]. Consistent with these findings, the selective proteasome inhibitor PS-519 prevents IκB degradation and inhibits downstream NF-κB signaling, reducing T cell activation in vitro and in vivo. Thus, it is effective in the treatment of psoriasis in the murine SCID-hu model [31]. In contrast, bortezomib, a different proteasome inhibitor, exacerbates symptoms in an imiquimod-induced psoriasis (IMQ) model [32]. Inhibition of proteasomes may induce a proinflammatory response, which manifests in increased cyclooxygenase-2 activity and prostaglandin E2 production [33]. The proteasome negatively regulates the transcription of inflammatory mediators regulated by the atypical, redox-dependent stimulus of the NF-κB pathway [34]. In addition, mutations in the proteasome subunits cause autoinflammatory disorders such as Nakajo-Nishimura syndrome and chronic atypical neutrophilic dermatosis [35].

Therefore, the study aimed to link the severity of oxidative stress (assessed by the level of lipid peroxidation and oxidative protein modifications) with the expression of the proteasomal system and autophagy in the blood cells of psoriasis patients.

## 2. Results

### 2.1. S Proteasome Subunit Expression and Activity in Blood Cells From Psoriatic Patients

The constitutive 20S proteasome contains three β subunits which perform caspase-like (β1), trypsin-like (β2), and chymotrypsin-like (β5) activities, whereas the immunoproteasome has three inducible subunits which exhibit chymotrypsin-like (β1i and β5i) and trypsin-like (β2i) activities.

**Figure 1:**
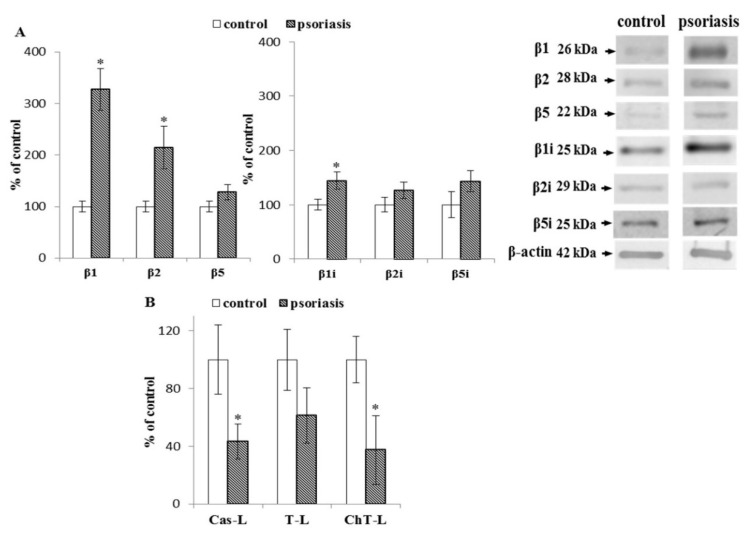
(**A**) Expression of the constitutive proteasome (β1, β2, β5) and immunoproteasome (β1i, β2i, β5i) subunits in lymphocytes from patients with psoriasis (*n* = 6), in comparison to healthy subjects (control), (*n* = 6). (**B**) Caspase-like (Cas-L), trypsin-like (T-L), and chymotrypsin-like (ChT-L) activities of the proteasomes in lymphocytes from patients with psoriasis (*n* = 16) and healthy subjects (control), (*n* = 12). Values in (**A**) and (**B**) are expressed as a percentage of control. ∗—statistical significance at *p* < 0.05 vs. control.

The development of psoriasis favored a significant increase in the expression of the constitutive catalytic subunits β1, β2, and β5 in lymphocytes. The most substantial increase (more than 3-fold) was in the expression of the β1 subunit (Figure 1). A significant increase in β1i subunit expression and a modest increase in the level of β2i and β5i subunits of the immunoproteasome were also observed. In contrast, we observed over 50% reduction in caspase-like activity, for which the β1 subunit is responsible. Moreover, both the chymotrypsin-like activity attributed to β5, β1i, and β5i subunits, as well as the trypsin-like activity associated with β2 and β2i subunits, were reduced (by approximately 60% and 30%, respectively) compared to the cells of healthy people.

**Figure 2:**
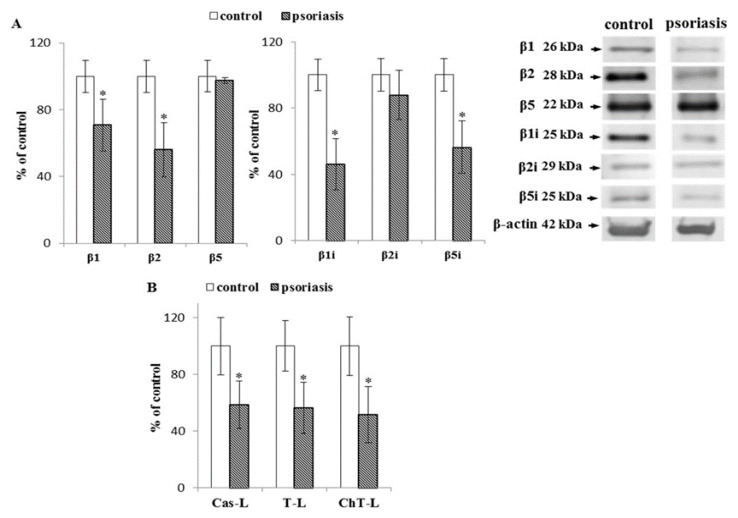
(**A**) Expression of the constitutive proteasome (β1, β2, β5) and immunoproteasome (β1i, β2i, β5i) subunits in granulocytes from patients with psoriasis (*n* = 6), in comparison to healthy subjects (control), (*n* = 6). (**B**) Caspase-like (Cas-L), trypsin-like (T-L), and chymotrypsin-like (ChT-L) activities of the proteasomes in granulocytes from patients with psoriasis (*n* = 16) and healthy subjects (control), (*n* = 12). Values in (**A**) and (**B**) are expressed as a percentage of control. ∗—statistical significance at *p* < 0.05 vs. control.

In the granulocytes of patients with psoriasis, the expression of constitutive subunits β1 and β2 and inducible subunits β1i and β5i was significantly reduced (50%) compared to healthy subjects (Figure 2). These changes were accompanied by a significant decrease (50%) in all three types of proteasomal activities (i.e., chymotrypsin-like, trypsin-like, caspase-like).

**Figure 3:**
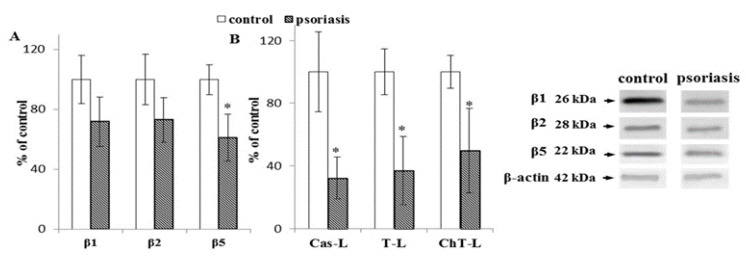
(**A**) Expression of the constitutive proteasome (β1, β2, β5) and immunoproteasome (β1i, β2i, β5i) subunits in erythrocytes from patients with psoriasis (*n* = 6), in comparison to healthy subjects (control), (*n* = 6). (**B**) Caspase-like (Cas-L), trypsin-like (T-L), and chymotrypsin-like (ChT-L) activities of the proteasomes in erythrocytes from patients with psoriasis (*n* = 16) and healthy subjects (control), (*n* = 12). Values in (**A**) and (**B**) are expressed as a percentage of control. ∗—statistical significance at *p* < 0.05 vs. control.

In contrast to lymphocytes and granulocytes, erythrocytes do not contain immunoproteasomes [36]. The expression of the 20S proteasome constitutive catalytic subunit β5 was significantly reduced (by about 30%), while β2 andβ1 showed only a slight reduction in erythrocytes of psoriasis patients (Figure 3) compared to healthy controls. All three 20S proteasome activities were significantly reduced (chymotrypsin-like by around 50%, trypsin-like by around 60%, and caspase-like by 70%).

### 2.2. Autophagy-Associated Protein Expression in Blood Cells From Psoriatic Patients

**Figure 4:**
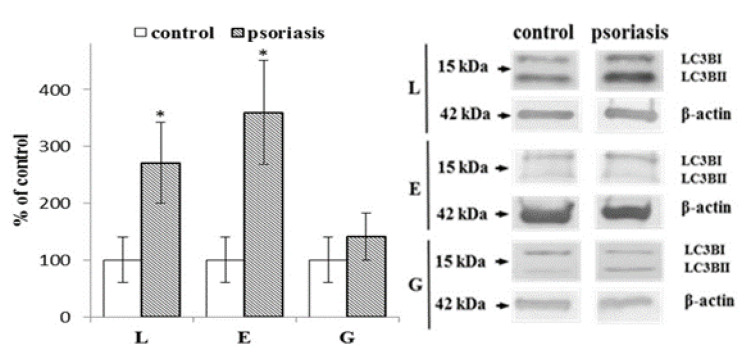
The ratio of LC3BII/LC3BI expression in lymphocytes (L), erythrocytes (E), and granulocytes (G) from psoriatic patients (*n* = 6), in comparison to healthy subjects (control), (*n* = 6). Values are expressed as a percentage of control. ∗—statistical significance at *p* < 0.05 vs. control.

The LC3B protein is an autophagy marker that has two forms (LC3B-I and LC3B-II). Following the activation of autophagy, LC3B is cleaved after the C-terminal glycine by ATG4 family proteins to form LC3-I and then conjugated with phosphatidylethanolamine to form LC3-II (Figure 4). Due to variability in the basal levels of LC3B in different cells, the ratio of LC3BII/LC3BI is used to assess the severity of autophagy. Lymphocytes and erythrocytes from patients with psoriasis show a significantly increased LC3BII/LC3BI ratio (about 3 and 4 times, respectively) compared to healthy individuals, while granulocytes have a similar LC3BII/LC3BI ratio in the control and psoriasis group.

**Figure 5:**
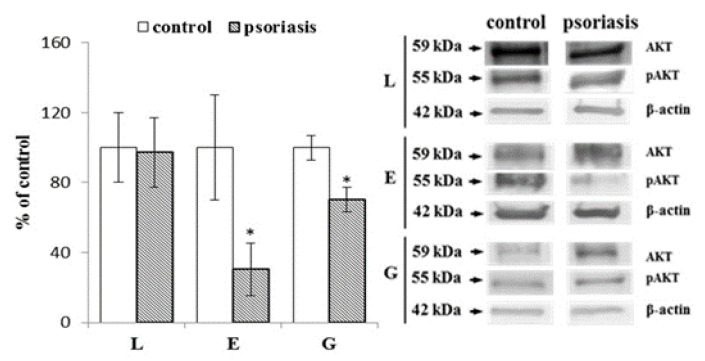
Phosphorylated AKT (pAKT)/AKT expression ratio in lymphocytes (L), erythrocytes (E), and granulocytes (G) from psoriatic patients (*n* = 6), in comparison to healthy subjects (control), (*n* = 6). Values are expressed as a percentage of control. ∗—statistical significance at *p* < 0.05 vs. control.

The serine-threonine kinase AKT plays a central role in the regulation of cell survival in a variety of diseases. AKT phosphorylates key molecules involved in the regulation of autophagy, and the AKT/mTOR pathway is one of the major activators of macroautophagy (Figure 5). Our data show a reduction in phosphorylation of AKT in erythrocytes and granulocytes (by about 70% and 30%, respectively) but not in lymphocytes of psoriatic patients.

### 2.3. Protein Modifications in Blood Cells From Psoriatic Patients

**Figure 6:**
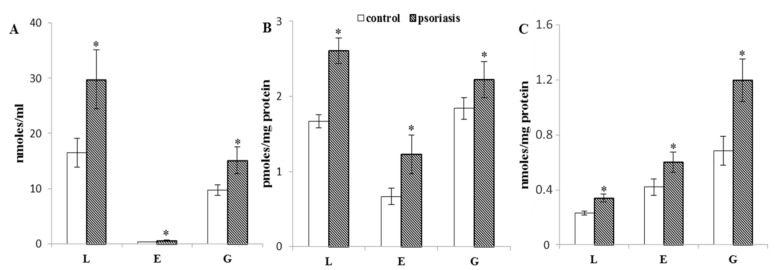
The level of 4-HNE (**A**), 4-HNE-protein adducts (**B**), and carbonylated proteins (**C**) in erythrocytes (E, *n* = 68), lymphocytes (L, *n* = 20), and granulocytes (G, *n* = 20) of psoriatic patients compared to healthy subjects (control) (E, *n* = 34, L, *n* = 10, and G, *n* = 10); ∗—statistical significance at *p* < 0.05 vs. control.

Oxidative stress can potentiate lipid peroxidation. One of the end products of lipid peroxidation is 4-HNE, which can make adducts with proteins. Lymphocytes, erythrocytes, and granulocytes from psoriatic patients have display 4-HNE concentrations that are two times higher than the blood cells from healthy subjects (Figure 6).

## 3. Discussion

Psoriasis is an inflammatory immune disease with a pathogenesis that is incompletely understood. However, it is clear that the development of this disease is accompanied by oxidative stress resulting from increased ROS production and disruption of antioxidant protection both local in skin cells and systemic in blood cells, as we have shown in previous studies [11,12]. Such conditions favor the oxidative modifications of the cellular components, including proteins, lipids, and DNA, and many studies have shown elevated levels of oxidative stress markers in serum/plasma and blood cells (erythrocytes, lymphocytes, and granulocytes) in patients with psoriasis [8,9,10,37]. In addition, a positive correlation between oxidative stress markers and psoriatic skin area and disease severity index has also been observed [15].

Under oxidative conditions, ROS interact directly with proteins, which causes both the oxidation of amino acid side chains and the breaking of peptide bonds to form carbonyl groups [38]. The results of this study demonstrate enhanced protein carbonylation in the blood cells of patients with psoriasis. Protein carbonylation is also a result of the reaction of proteins with reactive aldehydes, which are products of lipid peroxidation [16]. Phospholipids, especially their polyunsaturated fatty acids, are extremely susceptible to ROS, and as a result of oxidative fragmentation of hydrocarbon chains of these acids, reactive aldehydes are formed, including 4-HNE [39]. Elevated levels of 4-HNE were observed in the blood cells (erythrocytes, granulocytes, and lymphocytes) of patients with psoriasis, which confirms previous results showing the same effect in granulocytes and lymphocytes as well as plasma of patients [11,12].

4-HNE has two electrophilic centers and, therefore, can react with nucleophilic fragments of protein amino acid residues, mainly histidine, cysteine, and lysine, forming 4-HNE-protein adducts [12,40], whose elevated levels are observed in all examined blood cells of patients with psoriasis. The consequence of the formation of these adducts is a change in the structure of protein molecules, including the formation of intra- and intermolecular bonds. These structural changes can lead to protein cross-linking, which modifies the physicochemical and biological properties of proteins, as well as decreasing their susceptibility to degradation [40,41]. Protein carbonylation is an irreversible process, which means that damaged molecules are not repaired, and if they are not degraded, they can strongly disrupt cellular metabolism [42]. It has been shown that as a result of oxidative modification of protein molecules, their surface hydrophobicity increases, which is a key signal for the proteolytic system to recognize the protein and initiate its degradation [43].

Modified proteins are degraded mainly by the proteasomal systems (20S and 26S) [44]. However, during oxidative stress, the activity of the 26S proteasome decreases, while the activity of the 20S proteasome remains mostly unchanged [45]. Unsurprisingly, in many inflammatory diseases, dysregulation of proteasomal activity is observed [35,46]. Increased proteasome activity and expression but stable mRNA levels has been demonstrated in psoriatic skin cells, which has led to the suggestion that the expression of proteasomes and immunoproteasomes is post-transcriptionally regulated [30]. However, our study found a reduction in the caspase-, trypsin-, and chymotrypsin-like activities of the 20S proteasomes in blood cells of psoriatic patients, corresponding to increased levels of carbonylated proteins and 4-HNE-protein adducts. It is possible that under intense oxidative stress, even the 20S proteasome may not be sufficient to maintain efficient protein degradation. On the other hand, the 20S proteasome may be subject to oxidative modification [44], causing the level of carbonyl groups to negatively correlate with proteasome activity. This effect has been observed in HEK 293 cells [47]. It has also been shown that modifications of proteasome structure by 4-HNE, and as a result of carbonylation, S-glutathionylation, and glycoxidation, cause changes in the proteasome subunits, which affects 20S proteasomal activity [48].

The results of this study show that increased oxidative modifications of cellular proteins are accompanied by changes in the expression and activity of the catalytic subunits of the proteasome. Increased expression of both constitutive and immunoproteasome catalytic subunits in lymphocytes verifies that oxidative stress impacts proteasomes at the level of expression. The increase in proteasome expression under stress conditions is accompanied by oxidative modification of the associated proteins, leading to structural changes, consequently, reduced proteasomal activity. In addition, patients with psoriasis have previously been shown to have elevated levels of anti-proteasome antibodies in the blood [34], which may further reduce the activity of proteasomes, as demonstrated in in vitro studies [49].

The accumulation of proteins modified in the development of psoriasis may lead to the modification of signaling pathways. Such signaling pathways could include initiating inflammatory processes, which could lead to cell death [7]. Moreover, protein adducts have antigenic properties, which means that their proinflammatory effects may also be the result of the autoimmune response demonstrated in rheumatoid arthritis [50]. It is known that proinflammatory signaling pathways such as MAPK/AP-1, NF-κB, and JAK-STAT are involved in the development of psoriatic lesions [3,4,5]. Our previous studies have shown elevated levels of NF-κB and proinflammatory cytokines in plasma and the blood cells (granulocytes and lymphocytes) of patients with psoriasis [11,12]. However, adequate proteasomal activity is required to activate the canonical NF-κB pathway. NF-κB remains in the cytoplasm due to its interaction with the NF-κB inhibitor (IκB). When it is phosphorylated, IκB is degraded by the proteasome, and free NF-κB is transported to the cell nucleus, where it controls the transcription of proinflammatory genes [51]. However, the observed decrease in proteasomal activity may favor the inhibition of canonical activation of NF-κB (IκB phosphorylation-dependent) and may instead activate the unusual NF-κB pathway [52]. In our previous work, [11,13], we observed increased expression of Keap1, which is a cytosolic inhibitor of another transcription factor—Nrf2—in granulocytes and lymphocytes of patients with psoriasis, which may support the degradation of IκB [53]. Nrf2 causes the transcriptional activation of several 20S proteasomal subunits as well as the PA28αβ (11S)—proteasomal regulator [25] because the promoter regions of many 20S α and β subunits and PA28αβ subunits have electrophile/antioxidant response elements domains consisting of the RTGACnnGG core motif or the extended TMAnnRTGAYnnnGCAww motif [25]. Enhanced expression of Nrf-2 in lymphocytes of patients with psoriasis [13], induces the expression of constitutive catalytic subunits [54]. This may at least partly explain the increased protein expression of constitutive β1, β2, and β5 catalytic subunits in lymphocytes of patients with psoriasis observed in this study. It is also known that oxidative stress increases the expression of immunoproteasomes [26,55]. These findings may suggest that excessive release of inflammatory cytokines may cause alteration in the conversion of the proteasome to the immunoproteasome, which plays a role in the formation of peptides for presentation by MHC class I proteins [56]. On the other hand, immunoproteasomes have also been shown to be required to reduce inflammatory responses. However, our results also show a reduction of immunoproteasomal subunits in granulocytes.

In the degradation of damaged/modified proteins, the proteasomal system is supported by the process of autophagy, which degrades, among others, protein aggregates that have not undergone proteasomal proteolysis and may accumulate in cells [57,58]. Our findings indicate a significant increase in autophagy units in the blood cells of patients with psoriasis. In contrast to the proteasomal system, reactive oxygen species have been recognized as inducers of autophagy [59]. Therefore, increased oxidative stress in the blood cells of patients with psoriasis leads to increased expression of autophagic units, which in turn may also increase the degradation of 20S proteasome subunits, especially those that have undergone oxidative modifications. The severity of autophagy may be indicated by increased levels of phosphatidylethanolamines since it was previously found that the level of degradation products of these phospholipids (lysophosphatidylethanolamines) is reduced in psoriasis granulocytes [12]. In contrast, by coupling with LC3, phosphatidylethanolamines participate in the formation of the autophagosomes necessary to start the protein degradation process [60].

Finally, elevated levels of ROS observed during the development of psoriasis may suppress AKT phosphorylation, which, by inhibiting the mammalian target of rapamycin (mTOR) pathway, causes autophagy activation. This effect was observed in our study, especially in granulocytes and erythrocytes of patients with psoriasis. As a result, these actions weaken the inflammatory response and stimulate the catabolic program of protein and cell growth [61,62,63]. Given the significant increase in autophagy and the reduction in the number of erythrocytes and lymphocytes observed in patients with psoriasis [64,65], cell death may be the final result of autophagy in these cases. Additionally, it has been suggested that autophagy, like the proteasomal system, is closely related to the complex network of NF-κB and Nrf2 transcription factors [66]. Autophagy could be required for the full activation of NF-κB [67]. Therefore, several elements of the NF-κB system, including all IKK, NIK, and p62SQSTM1 subunits, are simultaneously autophagic substrates. Autophagic degradation of IκB appears to be needed as a prolonged response to NF-κB transcriptional activity [68]. Autophagy is also regulated by the Nrf2 transcription factor, which induces the expression of p62 (an autophagic sectosome 1 adapter) that interacts with the autophagic LC3 effector protein and is degraded by the autophagic lysosome [69]. Studies on cancer cells suggest that cells accumulate p62 as a result of inflammation [70]. We detected increased levels of both transcription factor Nrf2 and p62 in the blood cells of patients with psoriasis [11,13,71].

The results of this study show a decrease in the activity and efficiency of the proteasomal system in blood cells, which resulted in the accumulation of modified proteins e.g., in the form of 4-HNE-protein adducts and carbonylated proteins, which in turn can lead to modification the biological activity of proteasomes [72]. Moreover, it is known that in the case of psoriasis, an overproduction of chemoattractants by keratinocytes is observed, which results in increased migration of cells from the bloodstream to the skin [2]. For this reason, infiltration with immune cells is observed in the skin, what is an important element of psoriatic lesions and, consequently, the intensification of the keratinocyte-leukocyte interaction. In psoriasis, oxidative stress and elevated levels of cytokines in both the skin and blood are observed, suggesting that similar phenotypic changes occur in both peripheral blood leukocytes and skin infiltrating leukocytes. However oxidative stress results in changes in the conformation and activity of proteins involved in the regulation of transcription, modification and transmission of signaling molecules, both intracellular as well as cytokines, which modifies the interaction of immune cells with keratinocytes [2]. It was found that this applies especially to proteins participating in the antioxidant response, what additionally increases the oxidative stress in keratinocytes [11,12,16]. These changes are particularly important when modifying the Nrf2 transcription factor pathway, the increased activity of which has been described as a key factor in the proliferation of keratinocytes in psoriasis [73]. On the other hand, intensification of autophagy, especially in immune cells, most likely favors NF-κB activation [67], which results in an increase in the production of pro-inflammatory cytokines, which then have a paracrine or endocrine effect on keratinocytes, additionally strengthening the proinflammatory background of psoriasis development. Therefore, the observed changes suggest strengthening of proliferative and inflammatory signal by direct connection of processes taking place in blood cells, especially immune and epidermal cells [2]. It may be suggested that this is important metabolic information indicative of the appearance/existence of specific pathophysiological changes in keratinocytes and, consequently, may significantly support the early diagnosis of psoriasis.

In summary, the reduction of 20S proteasome activity in the blood cells of patients with psoriasis may be the result of the reduced expression of proteasome subunits. Additionally, structural modification of these proteins as a result of their interaction with 4-HNE or ROS could also affect proteasome activity. Concurrently, oxidative stress promotes impaired protein polyubiquitination and reduced degradation of proteins by the 26S proteasome [7]. Thus, although the development of psoriasis is accompanied by increased autophagy, the observed overexpression of total levels of carbonylated proteins and 4-HNE protein adducts indicates a lack of proteostatic balance in the blood cells of patients with psoriasis. We suggest that the use of antioxidant compounds in the treatment of psoriasis may support the maintenance of metabolic homeostasis in the blood cells of patients.

## 4. Materials and Methods

### 4.1. Patients Characterization

Blood samples were obtained from 32 female and 36 male psoriasis patients (total: 68 individuals, mean age: 38). The individuals had psoriasis for at least six months, with at least 10% of the total body surface area affected. The control group consisted of 15 healthy female and 19 healthy male individuals (total: 34 individuals, mean age: 38). The main exclusion criteria in all groups were the use of topical or oral medication four weeks before the start of the study, and the presence of any illnesses, smoking, or alcohol abuse. When analyzing the patient’s medical history, particular attention was paid to currently used drugs (antiplatelet and anticoagulant drugs) and comorbidities (circulatory, respiratory, liver, or kidney disease, as well as cancer and diabetes). The study was approved by the Local Bioethics Committee at the Medical University of Bialystok (Poland), No. R-I-002/289/2017 (approved on 28 September 2017). Written informed consent was obtained from all the patients.

### 4.2. Blood Samples

Blood samples were taken into tubes containing ethylenediaminetetraacetic acid (EDTA). Two-stage centrifugation was then carried out. In the first stage, the sample was centrifuged at 3000× *g* (4 °C) to obtain the plasma and the buffy coat. Erythrocytes, lymphocytes, and granulocytes were isolated from the buffy coat by gradient centrifugation using Gradisol G/L (1:5 ratio). Samples were layered on Gradisol and subjected to 25 min centrifugation at 300× *g* at room temperature. The individual cell fraction was collected, washed, and resuspended in PBS containing proteasome inhibitor mix. An antioxidant—butylhydroxytoluene (BHT)—was added to erythrocyte, lymphocyte, and granulocyte samples before storing them to prevent oxidation. The total protein content in the cells lysate was measured using a Bradford assay [74].

### 4.3. Proteasomal Activity

Blood cells were sonicated in lysis buffer containing 50 mM Tris-HCl (pH = 7.5), 1 mM EDTA, 1 mM ethylene-bis(oxyethylenenitrilo)tetraacetic acid EGTA, 0.5% Triton X, and centrifuged at 12,000× *g* for 15 min at 4 °C. The supernatants were diluted to 2 mg protein/mL and assayed for proteasome activity in the assay buffer containing 100 mM Tris/HCl pH = 7.5, 1 mM EDTA, 1 mM EGTA, using the chymotrypsin-like activity substrate, Suc-LLVY-7-amino-4-methylcoumarin (Suc-LLVY-AMC; Sigma-Aldrich, St. Louis, MO, USA), the trypsin-like activity substrate, Bz-VGR-AMC (Enzo Life Sciences, Inc., Farmingdale, NY, USA), or the caspase-like activity substrate, Z-LLE-AMC (Enzo Life Sciences, Inc., Farmingdale, NY, USA). All substrates were at a concentration of 100 μM in a final volume of 50 μL [75]. All components were added to a black 96 well plate and incubated at 37 °C for 30 min. The released fluorogenic 7-amino-4-methylcoumarin (AMC) concentration was measured spectrofluorometrically [355 nm excitation and 460 nm emission] using a fluorometric plate reader (EnSpire 2300 Multilabel Reader, PerkinElmer, Waltham, MA, USA). The amount of released AMC was evaluated from the AMC reference curve, which allowed expression of proteasome activity in pmol AMC/min/mg protein to be assessed. All tests were carried out in triplicate.

### 4.4. Protein Expression

Cell lysates were mixed with sample loading buffer (Laemmle buffer containing 5% 2-mercaptoethanol), heated at 95 °C for 10 min, and separated by 10% Tris-glycine SDS-PAGE. The same procedure was used to prepare the negative control (containing pure PBS buffer) and the positive control (commercially purchased complete cell lysate—Santa Cruz Biotechnology, Santa Cruz, CA, USA). β-actin was used as an internal loading control. Separated proteins were electrophoretically transferred onto nitrocellulose membranes. The blotted membranes were blocked with 5% skim milk in TBS-T buffer (5% Tween-20) for 1 h. Primary antibodies against β-actin (Sigma-Aldrich, St. Louis, MO, USA) and 20S β1, β1i, β2, β2i, β5, β5i subunits, LC3B, AKT, phosphorylated AKT (pAKT) (Enzo Life Sciences, Inc., Farmingdale, NY, USA) were used at a concentration of 1:1000. After incubation with primary antibodies, the membranes were then washed four times with TBS-T and incubated with goat or mouse or rabbit polyclonal alkaline phosphatase secondary antibodies (Sigma-Aldrich; St. Louis, MO, USA). Protein bands were visualized using the BCIP/NBT liquid substrate system (Sigma-Aldrich, St. Louis, MO, USA) and quantitated using the Versa Doc System and Quantity One software (Bio-Rad Laboratories Inc., Hercules, CA, USA). The results are expressed as a percentage of the expression determined in control cells.

### 4.5. Determination of 4-HNE Level

The product of phospholipid oxidative fragmentation—4-HNE-was measured by gas chromatography-mass spectrometry (GC-MS/MS GC-7000 quadrupole MS/MS, Agilent Technologies, Santa Clara, CA, USA). The selected ion monitoring (SIM) mode was used, as the *O*-PFB-oxime-TMS derivative, using minor modifications of the method of [76]. The following ions were used as follows: m/z 333.0 and 181.0 for 4-HNE-PFB-TMS, m/z 204.0.

### 4.6. Measurement of 4-HNE-Protein Adducts Level

The level of 4-HNE-protein adducts was measured using the ELISA method. Anti-4-HNE-His murine monoclonal antibody (genuine anti-4-HNE-His murine monoclonal antibody, clone 4-HNE 1g4) and goat anti-mouse antibody (Dako, Carpinteria, CA, USA) were used as primary and secondary antibodies, respectively. The concentrations of 4-HNE–protein adducts were normalized for 1 mg of protein.

### 4.7. Determination of Protein Carbonyl Groups Level

Protein oxidative modifications in plasma were estimated as the level of protein carbonyl groups by spectrophotometry (370 nm) using 2,4-dinitrophenylhydrazine [77] and were expressed as nmoles of carbonyl groups per mg of protein.

### 4.8. Statistical Analysis

An unpaired Student’s *t*-test was used to compare the mean values between conditions. Differences were considered significant when *p* < 0.05.

## Abbreviations

4-HNE	4-hydroxy-2,3-trans-nonenal
AKT	serine threonine kinase
AMC	7-amino-4-methylcoumarin
ATG4	autophagy-related 4 cysteine peptidase
BHT	butylhydroxytoluene
EDTA	ethylenediaminetetraacetic acid
EGTA	ethylene-bis(oxyethylenenitrilo)tetraacetic acid
ELISA	enzyme-linked immunosorbent assay
GC/MS	gas chromatography-mass spectrometry
IL	Interleukin
JAK-STAT	Janus kinase-signal transducer and activator of transcription pathway
LC3B	light chain 3B
MAPK/AP-1	mitogen-activated protein kinase associated protein 1
MHC	major histocompatibility complex
mTOR	mammalian target of rapamycin
NF-κB	nuclear factor kappa-light-chain-enhancer of activated B cells
Nrf2	nuclear factor erythroid 2-related factor 2
ROS	reactive oxygen species
SQSTM1	sequestosome 1
SIM	selected ion monitoring
